# Supplementary material for: Reliability of an interneuron response depends on an integrated sensory state
Source: eLife. 2019 Nov 13;8:e50566. doi: 10.7554/eLife.50566 (PMC6894930; doi:10.7554/eLife.50566)
Supplement: Supplementary file 1. — Both Chen et al. (2006) and Cook et al. (2019) are based on same collection of serial-section electron micrographs from White et al. (1986). Neurotransmitter information is based on Pereira et al. (2015). [file elife-50566-supp1.docx]

**Supplementary File 1. Presynaptic partners of AIA**

| Neuron Class | Pre  synaptic Neuron | Chen et al., 2006 | | Cook et al., 2019 | | | | Neuro-transmitter |
| --- | --- | --- | --- | --- | --- | --- | --- | --- |
|  |  | AIAL and AIAR | | AIAL | | AIAR | |  |
|  |  | Gap Junctions | Chemical Synapses | Gap Junctions | Chemical Synapses | Gap Junctions | Chemical Synapses |  |
| Sensory Neurons | ADFL | 1 |  |  | 1 |  |  | Acetylcholine + serotonin |
|  | ADFR |  |  |  |  | 3 |  |  |
|  | ADLL |  | 16 |  | 10 |  |  | Glutamate |
|  | ADLR |  |  |  |  |  | 11 |  |
|  | ASEL |  | 8 |  | 4 |  |  | Glutamate |
|  | ASER |  |  |  | 6 |  | 3 |  |
|  | ASGL |  | 19 |  | 15 |  |  | Glutamate |
|  | ASGR |  |  |  |  | 2 | 16 |  |
|  | ASHL |  | 17 |  | 13 |  |  | Glutamate |
|  | ASHR |  |  |  |  | 1 | 13 |  |
|  | ASIL | 4 | 4 | 1 | 1 |  |  | Orphan |
|  | ASIR |  |  | 1 | 1 | 1 | 3 |  |
|  | ASJL |  |  | 1 |  |  |  | Acetylcholine |
|  | ASJR |  |  |  |  |  |  |  |
|  | ASKL |  | 22 | 2 | 22 |  |  | Glutamate |
|  | ASKR |  |  |  | 1 | 1 | 25 |  |
|  | AWAL | 2 |  | 1 |  |  |  | Orphan |
|  | AWAR |  |  |  |  | 1 | 3 |  |
|  | AWBL |  |  | 1 | 2 |  |  | Acetylcholine |
|  | AWBR |  |  |  |  |  |  |  |
|  | AWCL |  | 8 |  | 2 |  | 3 | Glutamate |
|  | AWCR |  |  | 1 | 1 | 1 | 4 |  |
| Interneurons | ADAL |  |  |  | 1 |  |  | Glutamate |
|  | ADAR |  |  |  |  |  |  |  |
|  | AIAL | (N/A) | (N/A) |  |  | 1 |  | Acetylcholine |
|  | AIAR |  |  | 1 |  | 1 |  |  |
|  | AIBL |  |  | 2 |  |  |  | Glutamate |
|  | AIBR |  |  |  |  |  | 1 |  |
|  | AIML |  | 10 |  | 5 |  |  | Glutamate + serotonin |
|  | AIMR |  |  |  |  |  | 7 |  |
|  | AINL |  | 2 |  |  |  | 1 | Acetylcholine |
|  | AINR |  |  |  | 2 |  |  |  |
|  | AIZL |  | 4 |  | 8 |  |  | Glutamate |
|  | AIZR |  |  |  |  |  | 5 |  |
|  | AVFL |  |  |  |  |  |  | Orphan |
|  | AVFR |  |  |  | 1 |  |  |  |
|  | PVQL |  | 13 |  | 5 |  |  | Glutamate |
|  | PVQR |  |  |  | 1 |  | 10 |  |
| Motor Neurons | HSNL |  | 1 |  | 4 |  |  | Acetylcholine + serotonin |
|  | HSNR |  |  |  | 2 |  |  |  |
