# Supplementary material for: Reliability of an interneuron response depends on an integrated sensory state
Source: eLife. 2019 Nov 13;8:e50566. doi: 10.7554/eLife.50566 (PMC6894930; doi:10.7554/eLife.50566)
Supplement: Supplementary file 2. — Kolmogorov-Smirnov test statistics and sample sizes for all cumulative response time profiles presented, calculated for full 10 s stimulus pulse. Italics indicate non-WT genetic backgrounds. D test represents the maximum effect size across the distributions. p-values below 0.05 are bolded for emphasis. [file elife-50566-supp2.docx]

**Supplementary File 2. Cumulative Response Time Profiles, full data**

| **Test genotype or condition** | **Control genotype or condition** | **Neuron** | **Stimulus** | **Test # pulses  (# animals) [# experi-ments]** | **Control # pulses  (# animals)  [# experi-ments]** | **D test stat-istic** | **p-value** | **Figure** |
| --- | --- | --- | --- | --- | --- | --- | --- | --- |
| *AWA::Chr* response to odor after light pulses | WT, no *AWA::Chr* expression or pulses | AIA | 1.15 µM dia | 90 (47) [12] | 438 (226) [41] | 0.174 | **0.022** | 1S-3C |
| WT, 2^nd^ pulse | WT, 1^st^ pulse | AIA | AWA::Chr | 282 (282) [46] | 282 (282) [46] | 0.149 | **0.004** | 1S-3G |
| WT, 2^nd^ pulse | WT, 1^st^ pulse | AIA | 11.5 nM dia | 115 (115) [17] | 115 (115) [17] | 0.105 | 0.709 | 1S-3K |
| WT, 2^nd^ pulse | WT, 1^st^ pulse | AIA | 1.15 µM dia | 217 (217) [41] | 217 (217) [41] | 0.204 | **<0.001** | 1S-3O |
| *odr-7(ky4)* | WT | AIA | 1.15 µM dia | 70 (35) [5] | 78 (40) [8] | 0.465 | **<0.001** | 2A |
| *odr-10(ky32)* | WT | AIA | 1.15 µM dia | 32 (16) [3] | 78 (40) [8] | 0.700 | **<0.001** | 2A |
| *AWA::TeTx* | WT | AIA | 1.15 µM dia | 27 (14) [3] | 20 (10) [3] | 0.243 | 0.508 | 2C |
| *unc-7(e5)* | WT | AIA | 1.15 µM dia | 47 (24) [4] | 85 (43) [9] | 0.170 | 0.350 | 2D |
| *unc-9(fc16)* | WT | AIA | 1.15 µM dia | 58 (30) [5] | 85 (43) [9] | 0.104 | 0.851 | 2D |
| *unc-9(fc16) unc-7(e5)* | WT | AIA | 1.15 µM dia | 86 (43) [7] | 94 (47) [7] | 0.315 | **<0.001** | 2E |
| *unc-9(fc16) unc-7(e5)* | *unc-7(e5)* | AIA | 1.15 µM dia | 38 (19) [3] | 46 (23) [3] | 0.449 | **<0.001** | 2F |
| *unc-9(fc16) unc-7(e5); AWA,AIA::unc-9(WT)* | *unc-7(e5)* | AIA | 1.15 µM dia | 36 (18) [3] | 46 (23) [3] | 0.220 | 0.283 | 2F |
| *unc-9(fc16) unc-7(e5); AWA,AIA::unc-9(fc16)* | *unc-7(e5)* | AIA | 1.15 µM dia | 34 (17) [3] | 46 (23) [3] | 0.568 | **<0.001** | 2F |
| *unc-9(fc16) unc-7(e5)* | WT | AIA | AIA::Chr | 30 (15) [4] | 50 (25) [4] | 0.160 | 0.723 | 2H |
| *unc-9(fc16) unc-7(e5)* | WT | AWA | AIA::Chr | 48 (25) [4] | 58 (29) [4] | 0.096 | 0.968 | 2J |
| *AWA::TeTx* | WT | AIA | AWA::Chr | 56 (28) [4] | 146 (73) [9] | 0.158 | 0.264 | 2S-1A |
| *unc-9(fc16) unc-7(e5)* | WT | AIA | 11.5 nM dia | 61 (31) [4] | 70 (35) [4] | 0.329 | **0.002** | 2S-1B |
| *unc-9(fc16) unc-7(e5)* | WT | AIA | AWA::Chr | 96 (48) [6] | 146 (73) [9] | 0.204 | **0.016** | 2S-1D |
| *unc-18(e234)* | WT | AIA | AIA::Chr | 46 (24) [5] | 64 (32) [5] | 0.108 | 0.914 | 2S-1G |
| *unc-18(e234)* | WT | AWA | AIA::Chr | 58 (29) [4] | 69 (35) [4] | 0.343 | **0.001** | 2S-1I |
| *unc-18(e234)* | WT | AIA | 11.5 nM dia | 34 (17) [3] | 66 (33) [5] | 0.642 | **<0.001** | 3A |
| *unc-13(e51)* | WT | AIA | 11.5 nM dia | 24 (12) [2] | 66 (33) [5] | 0.527 | **0.001** | 3A |
| *unc-18(e234)* | WT | AIA | AWA::Chr | 57 (30) [6] | 146 (76) [16] | 0.427 | **<0.001** | 3C |
| *unc-18(e81)* | WT | AIA | AWA::Chr | 40 (20) [3] | 146 (76) [16] | 0.415 | **<0.001** | 3C |
| *unc-13(e51)* | WT | AIA | AWA::Chr | 45 (23) [4] | 146 (76) [16] | 0.321 | **0.002** | 3C |
| *unc-13(e51)* | WT | AWA | AWA::Chr | 36 (18) [2] | 74 (41) [5] | 0.088 | 0.992 | 3E |
| *unc-18(e234)* | WT | AIA | 1.15 µM dia | 56 (29) [5] | 145 (74) [13] | 0.290 | **0.002** | 3S-1A |
| *unc-18(e81)* | WT | AIA | 1.15 µM dia | 72 (36) [4] | 145 (74) [13] | 0.107 | 0.640 | 3S-1A |
| *unc-13(e51)* | WT | AIA | 1.15 µM dia | 46 (23) [4] | 145 (74) [13] | 0.180 | 0.208 | 3S-1A |
| *unc-31(e928)* | WT | AIA | AWA::Chr | 50 (25) [5] | 146 (76) [16] | 0.089 | 0.931 | 3S-1E |
| *unc-18(e234)* | *eat-4-FRT* | AIA | AWA::Chr | 154 (77) [12] | 214 (107) [16] | 0.264 | **<0.001** | 4C |
| *eat-4-FRT; AWC,ASE,ASK,ASG::nFLP* | *eat-4-FRT* | AIA | AWA::Chr | 64 (33) [6] | 214 (107) [16] | 0.360 | **<0.001** | 4C |
| *eat-4-FRT; ASK::nFLP* | *eat-4-FRT* | AIA | AWA::Chr | 80 (40) [6] | 214 (107) [16] | 0.224 | **0.006** | 4D |
| *eat-4-FRT; ASG::nFLP* | *eat-4-FRT* | AIA | AWA::Chr | 67 (34) [4] | 214 (107) [16] | 0.062 | 0.989 | 4E |
| *eat-4-FRT; AWC::nFLP* | *eat-4-FRT* | AIA | AWA::Chr | 123 (62) [10] | 214 (107) [16] | 0.129 | 0.147 | 4F |
| *eat-4-FRT; AWC,ASE::nFLP* | *eat-4-FRT* | AIA | AWA::Chr | 55 (29) [4] | 214 (107) [16] | 0.188 | 0.090 | 4G |
| *che-1(p674)* | WT | AIA | AWA::Chr | 96 (48) [8] | 48 (24) [4] | 0.188 | 0.211 | 4H |
| *unc-18(e234)* | WT | AIA | AWA::Chr | 70 (46) [5] | 80 (41) [5] | 0.521 | **<0.001** | 4I |
| *unc-18(e234); AWC,ASE::unc-18(e234)* | WT | AIA | AWA::Chr | 72 (36) [4] | 80 (41) [5] | 0.629 | **<0.001** | 4I |
| *unc-18(e234); AWC,ASE::unc-18(WT)#1* | WT | AIA | AWA::Chr | 42 (21) [3] | 80 (41) [5] | 0.115 | 0.860 | 4I |
| *unc-18(e234); AWC,ASE::unc-18(WT)#2* | WT | AIA | AWA::Chr | 74 (37) [5] | 80 (41) [5] | 0.088 | 0.928 | 4I |
| *eat-4-FRT* | WT | AIA | AWA::Chr | 214 (107) [16] | 149 (76) [12] | 0.081 | 0.605 | 4S-1B |
| *AWC,ASE,ASK,ASG::nFLP* | WT | AIA | AWA::Chr | 37 (19) [3] | 149 (76) [12] | 0.179 | 0.658 | 4S-1B |
| WT | WT | ASK | 0 vs. 11.5 nM dia | 82 (41) [5] | 115 (58) [7] | 0.301 | **0.004** | 5S-1E |
| WT | WT | ASK | 0 vs. 1.15 µM dia | 84 (42) [5] | 115 (58) [7] | 0.404 | **<0.001** | 5S-1E |
| WT | WT | AWC | 0 vs. 11.5 nM dia | 60 (30) [5] | 52 (30) [5] | 0.096 | 0.959 | 5S-1F |
| WT | WT | AWC | 0 vs. 1.15 µM dia | 60 (30) [5] | 52 (30) [5] | 0.276 | **0.029** | 5S-1F |
| WT | WT | ASE | 0 vs. 11.5 nM dia | 54 (27) [4] | 42 (21) [4] | 0.220 | 0.220 | 5S-1G |
| WT | WT | ASE | 0 vs. 1.15 µM dia | 53 (27) [4] | 42 (21) [4] | 0.307 | **0.024** | 5S-1G |
| *odr-7(ky4)* | WT | AIA | 0.9 µM IAA | 18 (9) [2] | 78 (41) [11] | 0.539 | **<0.001** | 5I |
| *ceh-36(ky640)* | WT | AIA | 0.9 µM IAA | 18 (9) [3] | 78 (41) [11] | 0.624 | **<0.001** | 5I |
| *odr-7(ky4) ceh-36(ky640)* | WT | AIA | 0.9 µM IAA | 28 (14) [3] | 78 (41) [11] | 0.571 | **<0.001** | 5I |
| *odr-7(ky4)* | WT | AIA | 9 µM IAA | 18 (9) [2] | 78 (41) [11] | 0.658 | **<0.001** | 5J |
| *ceh-36(ky640)* | WT | AIA | 9 µM IAA | 18 (9) [3] | 78 (41) [11] | 0.457 | **0.004** | 5J |
| *odr-7(ky4) ceh-36(ky640)* | WT | AIA | 9 µM IAA | 28 (14) [3] | 78 (41) [11] | 0.739 | **<0.001** | 5J |
| *odr-7(ky4)* | WT | AIA | 90 µM IAA | 18 (9) [2] | 77 (41) [11] | 0.687 | **<0.001** | 5K |
| *ceh-36(ky640)* | WT | AIA | 90 µM IAA | 17 (9) [3] | 77 (41) [11] | 0.237 | 0.416 | 5K |
| *odr-7(ky4) ceh-36(ky640)* | WT | AIA | 90 µM IAA | 28 (14) [3] | 77 (41) [11] | 0.796 | **<0.001** | 5K |
| WT | WT | AIA | 1.15 µM dia vs. 90 µM IAA | 77 (41) [11] IAA | 438 (226) [41] dia | 0.131 | 0.203 | 5L |
| WT | WT | AIA | 1.15 µM dia vs. OP50-conditioned medium | 39 (21) [4] OP50 | 438 (226) [41] dia | 0.147 | 0.420 | 5L |
| WT | WT | AWC | 0 vs. 0.9 µM IAA | 42 (21) [3] | 42 (21) [3] | 0.429 | **<0.001** | 5S-3D |
| WT | WT | AWC | 0 vs 9 µM IAA | 42 (21) [3] | 42 (21) [3] | 0.548 | **<0.001** | 5S-3D |
| WT | WT | AWC | 0 vs 90 µM IAA | 42 (21) [3] | 42 (21) [3] | 0.691 | **<0.001** | 5S-3D |
| WT | WT | AWA | 0 vs. 0.9 µM IAA | 80 (40) [5] | 78 (40) [5] | 0.292 | **0.002** | 5S-3E |
| WT | WT | AWA | 0 vs 9 µM IAA | 80 (40) [5] | 78 (40) [5] | 0.558 | **<0.001** | 5S-3E |
| WT | WT | AWA | 0 vs 90 µM IAA | 80 (40) [5] | 78 (40) [5] | 0.772 | **<0.001** | 5S-3E |
| WT | WT | ASK | 0 vs. 0.9 µM IAA | 60 (30) [3] | 60 (30) [3] | 0.510 | 0.510 | 5S-3F |
| WT | WT | ASK | 0 vs 9 µM IAA | 60 (30) [3] | 60 (30) [3] | 0.133 | 0.660 | 5S-3F |
| WT | WT | ASK | 0 vs 90 µM IAA | 60 (30) [3] | 60 (30) [3] | 0.167 | 0.375 | 5S-3F |
| WT | WT | AWA vs. AIA | 0.9 µM IAA | 80 (40) [5] AWA | 438 (226) [41] AIA | 0.807 | 0.103 | 5S-3G |
| WT | WT | AWA vs. AIA | 9 µM IAA | 80 (40) [5] AWA | 438 (226) [41] AIA | 0.677 | 0.115 | 5S-3H |
| WT | WT | AWA vs. AIA | 90 µM IAA | 80 (40) [5] AWA | 438 (226) [41] AIA | 0.125 | 0.189 | 5S-3I |
| WT | WT | ASK vs. AIA | 1.15 µM dia | 84 (42) [5] ASK | 438 (226) [41] AIA | 0.128 | 0.202 | 5S-3J |
