# Supplementary material for: Reliability of an interneuron response depends on an integrated sensory state
Source: eLife. 2019 Nov 13;8:e50566. doi: 10.7554/eLife.50566 (PMC6894930; doi:10.7554/eLife.50566)
Supplement: Supplementary file 3. — Magnitudes of responses to various stimuli, with either an unpaired t-test (if the number of comparisons is one) or an ordinary one-way ANOVA with Dunnett’s multiple comparisons test (if the number of comparisons exceeds one); * indicates paired t-test. Bolded genotype or stimulus indicates the control group used for comparisons. Italics indicate non-wildtype genetic background. p-values below 0.05 are bolded for emphasis. [file elife-50566-supp3.docx]

**Supplementary File 3. Calcium Response Magnitude Comparisons**

| **Neuron** | **Stimulus** | **All vs. Responses Only (R)** | **Genotype** | **Mean ± SEM** | **n** | **# compar-isons** | **p-value** | **95% Confidence Interval of Difference** | **Figure(s)** |
| --- | --- | --- | --- | --- | --- | --- | --- | --- | --- |
| AWA | AWA::Chr | R | **WT** | 129.0 ± 9.8 | 59 | 2 | **0.032** | [2.7, 69.1] | 3S-1D |
|  |  |  | *unc-13(e51)* | 93.1 ± 9.5 | 32 |  |  |  |  |
| ASK | **0** | All | WT | -4.0 ± 2.5 | 115 | 2 |  |  | 5S-1A |
|  | 11.5 nM dia |  |  | -20.8 ± 2.2 | 82 |  | **<0.001** | [17.2, 32.3] |  |
|  | 1.15 µM dia |  |  | -38.4 ± 2.2 | 84 |  | **<0.001** | [34.9, 49.9] |  |
| AWC | **0** | All | WT | 0.2 ± 2.0 | 52 | 2 |  |  | 5S-1B |
|  | 11.5 nM dia |  |  | 1.1 ± 1.9 | 60 |  | 0.921 | [-6.7, 5.0] |  |
|  | 1.15 µM dia |  |  | -7.8 ± 1.6 | 60 |  | **0.005** | [2.2, 13.9] |  |
| ASE | **0** | All | WT | 0.4 ± 0.7 | 42 | 2 |  |  | 5S-1C |
|  | 11.5 nM dia |  |  | 1.5 ± 0.8 | 54 |  | 0.743 | [-5.0, 2.8] |  |
|  | 1.15 µM dia |  |  | 6.8 ± 1.7 | 53 |  | **<0.001** | [-10.4, -2.6] |  |
| ASH | **0** | All | WT | -0.1 ± 0.5 | 78 | 2 |  |  | 5S-1D |
|  | 1.15 µM dia |  |  | -0.5 ± 0.7 | 86 |  | 0.998 | [-14.4, 15.0] |  |
|  | +100 mM NaCl |  |  | 76.5 ± 7.7 | 86 |  | **<0.001** | [-91.3, -62.0] |  |
| ASK | **0** | All | WT | 3.4 ± 2.5 | 68 | 1 | **<0.001** | [-49.6, -36.4] | 5S-1I |
|  | 1.15 µM dia |  |  | -39.6 ± 2.2 | 92 |  |  |  |  |
|  | **0** |  | *unc-18(e234)* | -3.4 ± 3.2 | 12 | 1 | **<0.001** | [-42.3, -19.2] |  |
|  | 1.15 µM dia |  |  | -34.1 ± 3.7 | 24 |  |  |  |  |
|  | **0** |  | *odr-10(ky32)* | 12.8 ± 3.9 | 34 | 1 | **<0.001** | [-61.0, -41.6] |  |
|  | 1.15 µM dia |  |  | -38.5 ± 2.9 | 34 |  |  |  |  |
|  | 1.15 µM dia, compare to **WT** | | *unc-18(e234)* | | | 2 | 0.396 | [-15.7, 4.7] | 5S-1I |
|  |  |  | *odr-10(ky32)* | | |  | 0.952 | [-10.1, 7.9] |  |
| AWC | **0** | All | WT | -4.3 ± 2.1 | 34 | 1 | **0.002** | [-15.6, -3.5] | 5S-1J |
|  | 1.15 µM dia |  |  | -13.8 ± 2.1 | 53 |  |  |  |  |
|  | **0** |  | *unc-18(e234)* | -3.6 ± 1.2 | 10 | 1 | **<0.001** | [-20.1, -5.9] |  |
|  | 1.15 µM dia |  |  | -16.6 ± 2.1 | 26 |  |  |  |  |
|  | **0** |  | *odr-10(ky32)* | -5.2 ± 1.5 | 40 | 1 | **<0.001** | [-18.6, -8.5] |  |
|  | 1.15 µM dia |  |  | -18.8 ± 2.1 | 30 |  |  |  |  |
|  | 1.15 µM dia, compare to **WT** | | *unc-18(e234)* | | | 2 | 0.604 | [-4.4, 9.9] | 5S-1J |
|  |  |  | *odr-10(ky32)* | | |  | 0.191 | [-1.9, 11.8] |  |
| ASE | **0** | All | WT | 0.2 ± 0.8 | 38 | 1 | **<0.001** | [10.5, 23.3] | 5S-1K |
|  | 1.15 µM dia |  |  | 17.0 ± 2.4 | 64 |  |  |  |  |
|  | **0** |  | *unc-18(e234)* | -1.3 ± 0.7 | 40 | 1 | 0.074 | [-0.2, 4.7] |  |
|  | M dia |  |  | 0.9 ± 0.96 | 44 |  |  |  |  |
|  | **0** |  | *odr-10(ky32)* | -0.9 ± 0.9 | 26 | 1 | **<0.001** | [13.9, 25.9] |  |
|  | 1.15 µM dia |  |  | 19.0 ± 2.5 | 38 |  |  |  |  |
|  | 1.15 µM dia, compare to **WT** | | *unc-18(e234)* | | | 2 | **<0.001** | [9.4, 22.9] | 5S-1K |
|  |  |  | *odr-10(ky32)* | | |  | 0.777 | [-9.1, 5.2] |  |
| ASK | **--** | All | WT | 1.1 ± 3.0 | 24 | 1* | 0.620 | [-12.9, 7.9] | 5S-1O |
|  | AWA::Chr |  |  | -1.5 ± 3.1 | 24 |  |  |  |  |
| AWC | **--** | All | WT | 1.3 ± 1.9 | 44 | 1* | 0.899 | [-9.8, 8.6] | 5S-1O |
|  | AWA::Chr |  |  | 0.8 ± 4.4 | 44 |  |  |  |  |
| ASE | **--** | All | WT | 0.4 ± 1.3 | 44 | 1* | 0.113 | [-0.9, 8.2] | 5S-1O |
|  | AWA::Chr |  |  | 4.0 ± 1.3 | 44 |  |  |  |  |
| AWC | **0** | All | WT | -0.3 ± 2.4 | 42 | 3 |  |  | 5S-3A |
|  | 0.9 µM IAA |  |  | -19.7 ± 1.9 | 42 |  | **<0.001** | [12.0, 26.9] |  |
|  | 9 µM IAA |  |  | -18.2 ± 2.4 | 42 |  | **<0.001** | [10.5, 25.3] |  |
|  | 90 µM IAA |  |  | -16.8± 2.1 | 42 |  | **<0.001** | [9.1, 23.9] |  |
| AWA | **0** | All | WT | 14.2 ± 1.6 | 80 | 3 |  |  | 5S-3B |
|  | 0.9 µM IAA |  |  | 20.9 ± 2.2 | 78 |  | 0.766 | [-26.4, 13.1] |  |
|  | 9 µM IAA |  |  | 81.4 ± 8.0 | 78 |  | **<0.001** | [-86.9, -47.4] |  |
|  | 90 µM IAA |  |  | 185.8 ± 8.4 | 78 |  | **<0.001** | [-191.3,  -151.8] |  |
| ASK | **0** | All | WT | 0.9 ± 2.2 | 60 | 3 |  |  | 5S-3C |
|  | 0.9 µM IAA |  |  | -2.4 ± 1.8 | 60 |  | 0.586 | [-4.1, 10.8] |  |
|  | 9 µM IAA |  |  | 1.4 ± 2.2 | 60 |  | 0.996 | [-8.0, 6.9] |  |
|  | 90 µM IAA |  |  | 10.1 ± 2.6 | 60 |  | **0.011** | [-16.7, -1.8] |  |
| AIA | **1.15 µM dia** | R | WT | 94.9 ±3.0 | 404 | 3 |  |  | 5S-3L |
|  | 90 µM IAA |  |  | 107.2 ± 4.2 | 77 |  | 0.211 | [-28.9, 4.3] |  |
|  | *E. coli* OP50-conditioned medium |  |  | 105.8 ± 8.9 | 37 |  | 0.581 | [-33.9, 12.1] |  |
|  | AWA::Chr |  |  | 87.0 ± 3.0 | 321 |  | 0.166 | [-2.1, 17.9] |  |
| AIA | 1.15 µM dia | R | **WT** | 83.2 ± 4.4 | 73 | 3 |  |  | 4S-2D |
|  |  |  | *odr-7(ky4)* | 51.7 ± 4.8 | 42 |  | **<0.001** | [15.6, 47.5] |  |
|  |  |  | *odr-10(ky32)* | 54.3 ± 7.9 | 15 |  | **0.010** | [5.6, 52.3] |  |
| AIA | 1.15 µM dia | R | **WT** | 126.7 ± 18.0 | 18 | 1 |  |  | 4S-2E |
|  |  |  | *AWA::TeTx* | 102.0 ± 12.0 | 26 |  | 0.240 | [-66.6, 17.1] |  |
| AIA | AWA::Chr | R | **WT** | 80.7 ± 6.0 | 88 | 4 |  |  | 4S-2F, 4S-2H |
|  |  |  | AWA::TeTx | 94.8 ± 10.9 | 32 |  | 0.709 | [-46.6, 18.3] | 4S-2F |
|  |  |  | *unc-7(e5) unc-9(fc16)* | 79.6 ± 8.4 | 43 |  | >0.999 | [-28.2, 30.3] | 4S-2H |
| AIA | 11.5 nM dia | R | **WT** | 58.8 ± 6.0 | 49 | 1 |  |  | 4S-2G |
|  |  |  | *unc-7(e5) unc-9(fc16)* | 36.4 ± 5.8 | 26 |  | **0.018** | [-40.8, -4.0] |  |
| AIA | 1.15 µM dia | R | ***unc-7(e5)*** | 113.9 ± 9.6 | 42 | 3 |  |  | 4S-2I |
|  |  |  | *unc-7(e5) unc-9(fc16)* | 35.3 ± 5.0 | 22 |  | **<0.001** | [47.6, 107.7] |  |
|  |  |  | *unc-7(e5) unc-9(fc16); AWA,AIA::unc-9(WT)* | 64.6 ± 7.8 | 28 |  | **<0.001** | [22.4, 76.2] |  |
|  |  |  | *unc-7(e5) unc-9(fc16); AWA,AIA::unc-9(fc16)* | 38.8 ± 4.5 | 17 |  | **<0.001** | [43.4, 106.8] |  |
| AIA | 11.5 nM dia | R | **WT** | 94.4 ± 7.4 | 59 | 2 |  |  | 4S-2J |
|  |  |  | *unc-13(e51)* | 77.6 ± 6.7 | 21 |  | 0.366 | [-13.3, 46.7] |  |
|  |  |  | *unc-18(e234)* | 98.9 ± 9.7 | 32 |  | 0.901 | [-30.4, 21.4] |  |
| AIA | 1.15 µM dia | R | **WT** | 98.9 ± 7.2 | 134 | 5 |  |  | 4S-2K |
|  |  |  | *unc-13(e51)* | 82.6 ± 7.2 | 36 |  | 0.442 | [-10.4, 43.0] |  |
|  |  |  | *unc-18(e234)* | 87.6 ± 8.3 | 53 |  | 0.667 | [-11.8, 34.3] |  |
|  |  |  | *unc-18(e81)* | 97.4 ± 5.7 | 66 |  | >0.999 | [-19.8, 22.9] |  |
| AIA | AWA::Chr | R | **WT** | 107.2 ± 6.8 | 80 | 10 |  |  | 4S-2L |
|  |  |  | *unc-13(e51)* | 106.1 ± 7.0 | 33 |  | >0.999 | [-33.6, 36.0] |  |
|  |  |  | *unc-18(e234)* | 130.7 ± 8.7 | 50 |  | 0.248 | [-53.7, 6.9] |  |
|  |  |  | *unc-18(e81)* | 98.3 ± 7.2 | 35 |  | 0.996 | [-25.2, 43.0] |  |
| AIA | AWA::Chr | R | ***eat-4-FRT*** | 79.9 ± 5.7 | 128 | 5 |  |  | 4S-2N |
|  |  |  | *eat-4-FRT; AWC,ASE,ASK,ASG::nFLP* | 90.5 ± 8.2 | 50 |  | 0.781 | [-35.7, 14.6] |  |
|  |  |  | *eat-4-FRT; AWC,ASE::nFLP* | 82.1 ± 8.2 | 31 |  | >0.999 | [-32.4, 28.0] |  |
|  |  |  | *eat-4-FRT;ASK::nFLP* | 78.1 ± 6.1 | 58 |  | >0.999 | [-22.1, 25.6] |  |
|  |  |  | *eat-4-FRT; AWC::nFLP* | 86.3 ± 6.8 | 72 |  | 0.944 | [-28.6, 15.8] |  |
|  |  |  | *eat-4-FRT; ASG::nFLP* | 115.5 ± 10.0 | 43 |  | **0.003** | [-62.2, -9.1] |  |
| AIA | AWA::Chr | R | **WT** | 78.4 ± 4.2 | 84 | 3 |  |  | 4S-2O |
|  |  |  | *unc-18(e234)* | 81.3 ± 3.7 | 125 |  | 0.961 | [-19.9, 14.2] |  |
|  |  |  | *eat-4-FRT* | 79.9 ± 5.7 | 128 |  | 0.994 | [-18.4, 15.5] |  |
|  |  |  | WT*; AWC,ASE,ASK,ASG::nFLP* | 91.4 ± 11.8 | 21 |  | 0.602 | [-42.5, 16.4] |  |
| AIA | AWA::Chr | R | **WT** | 75.8 ± 7.7 | 27 | 1 |  |  | 4S-2P |
|  |  |  | *che-1(p674)* | 69.7 ± 8.2 | 49 |  | 0.625 | [-30.9, 18.7] |  |
