# Supplementary material for: Reliability of an interneuron response depends on an integrated sensory state
Source: eLife. 2019 Nov 13;8:e50566. doi: 10.7554/eLife.50566 (PMC6894930; doi:10.7554/eLife.50566)
Supplement: Supplementary file 4. — Rise times (t66-t33) of responses to various stimuli, with either an ordinary one-way ANOVA with Dunnett’s multiple comparisons test. Bolded genotype or stimulus indicates the control group used for comparisons. Italics indicate non-wildtype genetic background. [file elife-50566-supp4.docx]

**Supplementary File 4. Calcium Rise Time Comparisons**

| **Neuron** | **Stimulus** | **Genotype** | **Mean ± SEM** | **n** | **# comparisons** | **p-value** | **95% Confidence Interval** | **Figure** |
| --- | --- | --- | --- | --- | --- | --- | --- | --- |
| AIA | **1.15 µM dia** | WT | 0.40 ± 0.02 s | 390 | 3 |  |  | 5S-3M |
|  | 90 µM IAA |  | 0.30 ± 0.05 s | 76 |  | 0.332 | [-0.05, 0.24] |  |
|  | *E. coli* OP50-conditioned medium |  | 0.46 ± 0.09 s | 37 |  | 0.837 | [-0.26, 0.14] |  |
|  | AWA::Chr |  | 0.41 ± 0.03 s | 260 |  | 0.993 | [-0.10, 0.08] |  |
| AIA | AWA::Chr | **WT** | 0.41 ± 0.03 s | 260 | 3 |  |  | 4S-2M |
|  |  | *unc-13(e51)* | 0.33 ± 0.05 s | 29 |  | 0.824 | [-0.15, 0.29] |  |
|  |  | *unc-18(e234)* | 0.38 ± 0.03 s | 251 |  | 0.866 | [-0.07, -0.13] |  |
|  |  | *unc-18(e81)* | 0.21 ± 0.05 s | 29 |  | 0.111 | [-0.03, 0.41] |  |
