# Supplementary material for: Reliability of an interneuron response depends on an integrated sensory state
Source: eLife. 2019 Nov 13;8:e50566. doi: 10.7554/eLife.50566 (PMC6894930; doi:10.7554/eLife.50566)
Supplement: Supplementary file 5. [file elife-50566-supp5.docx]

**Supplementary File 5. Strain List**

| **Strain Name** | **Description** | **Genotype** | **Figure(s)** |
| --- | --- | --- | --- |
| CX14647 | *AWA::GCaMP2.2b* | *kyIs587* [*gpa-6p::GCaMP2.2b* 50ng/µl, *unc-122p::dsRed* 15ng/µl, *pSM* 35ng/µl, integrated by UV] | 1A, 1C, 1F, 1S-1BtoD, 1S-3B, 3G, 5G, 5S-3B, 5S-3E, 5S-3GtoJ |
| CX16573 | *AWA::Chr (AWA::Chrimson; AWA::GCaMP2.2b)* | *kyEx5662* [*odr-7p::Chrimson::sl2::mCherry* 5ng/µl, *elt-2p::mCherry* 2ng/µl, *pSM* 93ng/µl]; *kyIs587* | 1A, 1C, 1F, 1S-1BtoF, 1S-3A, 1S-3B, 3E, 3G, 3S-1C, 3S-1D |
| CX15257 | *AIA::GCaMP5A* | *kyEx5128* [*gcy-28dp::GCaMP5A*, *unc-122::dsRed* 15ng/µl] | 1B, 1DtoF, 1S-2AtoD, 1S-3B, 1S-3C, 1S-3KtoR, 2AtoE, 2S-1BtoC, 3A, 3G, 3S-1A, 4S-2BtoE, 4S-2G, 4S-2ItoK, 5ItoL, 5S-3GtoM, 6AtoC, 6S-1AtoB |
| CX16561 | *AWA::Chr (AWA::Chrimson; AIA::GCaMP5A)* | *kyEx5662; kyEx5128* | 1B, 1DtoF, 1S-2AtoD, 1S-3AtoJ, 2S-1A, 2S-1D, 3BtoD, 3G, 3S-1B, 3S-1E, 4H, 4I, 4S-1AtoD, 4S-2A, 4S-2F, 4S-2H, 4S-2LtoM, 4S-2OtoP, 5S-3L, 5S-3M |
| CX16171 | *odr-7; AIA::GCaMP5A* | *odr-7(ky4)X; kyEx5128* | 2A, 2B, 4S-2D, 5ItoK, 5S-3K |
| CX16170 | *odr-10; AIA::GCaMP5A* | *odr-10(ky32)X; kyEx5128* | 2A, 2B, 4S-2D |
| CX16584 | *AWA::TetanusToxin; AIA::GCaMP5A* | *kyEx3848* [*gpa-6p::TeTx* 20ng/µl, *elt-2p::mCherry* 2ng/µl]; *kyEx5128* | 2C, 4S-2E |
| CX17519 | *AWA::TetanusToxin; AWA::Chrimson; AIA::GCaMP5A* | *kyEx6140* [*gpa-6p::TeTx::sl2::mCherry* 20ng/µl, *myo-2p::mCherry* 0.5ng/µl]; *kyEx5662; kyEx5128* | 2S-1A, 4S-2F |
| CX18039 | *unc-7(e5); AIA::GCaMP5A* | *unc-7(e5); kyEx5128* | 2D, 2F, 2S-1E, 4S-2I |
| CX16980 | *unc-9(fc16); AIA::GCaMP5A* | *unc-9(fc16); kyEx5128* | 2D |
| CX16979 | *unc-9 unc-7; AIA::GCaMP5A* | *unc-9(fc16) unc-7(e5)(X); kyEx5128* | 2E, 2F, 2S-1B, 2S-1C, 2S-1E, 4S-2G, 4S-2I |
| CX18040 | *unc-9 unc-7; AWA,AIA::unc-9(fc16); AIA::GCaMP5A* | *unc-9(fc16) unc-7(e5)(X);* [*gpa-6p::unc-9(fc16)* 25ng/µl, *ins-1(s)p::unc-9(fc16)::sl2::tagRFP* 20ng/µl, *unc-122p::GFP* 15ng/µl]*; kyEx5128* | 2F, 2S-1E, 4S-2I |
| CX18041 | *unc-9 unc-7; AWA,AIA::unc-9(WT); AIA::GCaMP5A* | *unc-9(fc16) unc-7(e5)(X);* [*gpa-6p::unc-9(WTgenomic)* 25ng/µl, *ins-1(s)p::unc-9(WTgenomic)::sl2::tagRFP* 20ng/µl, *unc-122p::GFP* 15ng/µl]*; kyEx5128* | 2F, 2S-1E, 4S-2I |
| CX17320 | *unc-9 unc-7; AWA::Chrimson; AIA::GCaMP5A* | *unc-9(fc16) unc-7(e5)X; kyEx5662; kyEx5128* | 2S-1D, 4S-2H |
| CX17432 | *AIA::Chr (AIA::Chrimson; AIA::GCaMP5A)* | *kyEx6105* [*ins-1(s)p::Chrimson::sl2::mCherry* 20ng/µl, *elt-2p::mCherry* 2ng/µl]; *kyEx5128* | 2G, 2H, 2S-1F, 2S-1G |
| CX17895 | *unc-9 unc-7; AIA::Chrimson; AIA::GCaMP5A* | *unc-9(fc16) unc-7(e5)X; kyEx6105; kyEx5128* | 2G, 2H |
| CX17464 | *AIA::Chr (AIA::Chrimson; AWA::GCaMP2.2b)* | *kyEx6105; kyIs598* | 2I, 2J, 2S-1H, 2S-1I |
| CX17897 | *unc-9 unc-7; AIA::Chrimson; AWA::GCaMP2.2b* | *unc-9(fc16) unc-7(e5)X; kyEx6105; kyEx3225* | 2I, 2J |
| CX17584 | *unc-18; AIA::Chrimson; AIA::GCaMP5A* | *unc-18(e234)X; kyEx6105; kyEx5128* | 2S-1F, 2S-1G |
| CX17640 | *unc-18; AIA::Chrimson; AWA::GCaMP2.2b* | *unc-18(e234)X; kyEx6105; kyIs598* | 2S-1H, 2S-1I |
| CX16591 | *unc-13; AIA::GCaMP5A* | *unc-13(e51)I; kyEx5128* | 3A, 3G, 3S-1A, 4S-2J, 4S-2K |
| CX16412 | *unc-18; AIA::GCaMP5A* | *unc-18(e234)X; kyEx5128* | 3A, 3S-1A, 4S-2J, 4S-2K |
| CX16592 | *unc-13; AWA::Chrimson; AIA::GCaMP5A* | *unc-13(e51)I; kyEx5662; kyEx5128* | 3C, 3F, 3G, 4S-2L, 4S-2M |
| CX17158 | *unc-18; AWA::Chrimson; AIA::GCaMP5A* | *unc-18(e234)X; kyEx5662; kyEx5128* | 3BtoD, 3S-1B, 4C, 4I, 4S-1A, 4S-1D, 4S-2L, 4S-2M, 4S-2O |
| CX17640 | *unc-18(e81); AWA::Chrimson; AIA::GCaMP5A* | *unc-18(e81)X; kyEx5662; kyEx5128* | 3C, 3S-1A, 4S-2KtoM |
| CX17213 | *unc-13; AWA::Chrimson; AWA::GCaMP2.2b* | *unc-13(e51)I; kyEx5662; kyIs598* | 3EtoG, 3S-1C, 3S-1D |
| CX17319 | *unc-31; AWA::Chrimson; AIA::GCaMP5A* | *unc-31(e928)IV; kyEx5662; kyEx5128* | 3S-1E |
| CX17714 | *eat-4-FRT*; *coinjection marker control;* *AWA::Chrimson; AIA::GCaMP5A* | *kySi76* [*let-85UTR::FRT::mCherry* after *eat-4* endogenous stop codon] *kySi77* [FRT before eat-4 endogenous start codon] III; *kyEx6183* [*elt-2p::nlsGFP* 2ng/µl]; *kyEx5662; kyEx5128* | 4CtoG, 4S-1A, 4S-1B, 4S-2N, 4S-2O |
| CX17679 | *eat-4-FRT; AWC,ASE,ASK,ASG::nFlippase; AWA::Chrimson; AIA::GCaMP5A* | *kySi76* *kySi77 III; kyEx6153* [*tax-4p::nFlippase* 40ng/µl, *elt-2p::nlsGFP* 2ng/µl, *pSM* 58ng/µl]; *kyEx5662; kyEx5128* | 4CtoG, 4S-1A, 4S-2N |
| CX17722 | *eat-4-FRT; ASK::nFlippase; AWA::Chrimson; AIA::GCaMP5A* | *kySi76 kySi77 III; kyEx6150* [*sra-9p::nFlippase* 40ng/µl, *elt-2p::nlsGFP* 2ng/µl, *pSM* 58 ng/µl]; *kyEx5662; kyEx5128* | 4D, 4S-1A, 4S-2N |
| CX17892 | *eat-4-FRT; ASG::nFlippase; AWA::Chrimson; AIA::GCaMP5A* | *kySi76 kySi77 III; kyEx6169* [*gcy-15p::nFlippase* 25ng/µl, *elt-2p::nlsGFP* 2ng/µl, *pSM* 73ng/µl]; *kyEx5662; kyEx5128* | 4E, 4S-1A, 4S-2N |
| CX17611 | *eat-4-FRT; AWC::nFlippase; AWA::Chrimson; AIA::GCaMP5A* | *kySi76 kySi77 III; kyEx6242* [*odr-1p::nFlippase* 5ng/µl, *elt-2p::nlsGFP* 2ng/µl, *pSM* 93ng/µl]; *kyEx5662; kyEx5128* | 4F, 4S-1A, 4S-2N |
| CX17723 | *eat-4-FRT; AWC,ASE::nFlippase; AWA::Chrimson; AIA::GCaMP5A* | *kySi76 kySi77 III; kyEx6240* [*ceh-36p::nFlippase* 15ng/µl, *elt-2p::nlsGFP* 2ng/µl, *pSM* 83ng/µl]; *kyEx5662; kyEx5128* | 4G, 4S-1A, 4S-2N |
| CX17678 | *che-1; AWA::Chrimson; AIA::GCaMP5A* | *che-1(p674)I; kyEx5662; kyEx5128* | 4H, 4S-1C, 4S-2P |
| CX17866 | *AWC,ASE,ASK,ASG::nFlippase; AWA::Chrimson; AIA::GCaMP5A* | *kyEx6153; kyEx5662; kyEx5128* | S4-1A, S4-1B, 4S-2O |
| CX17675 | *unc-18; AWC,ASE::unc-18(WT)Rescue; AWA::Chrimson; AIA::GCaMP5A* (line A) | *unc-18(e234)X; kyEx6214* [*ceh-36p::unc-18(WTgenomic)::sl2::tagRFP* 15ng/µl, *unc-122p::GFP* 15ng/µl, *pSM* 70ng/µl]; *kyEx5662; kyEx5128* | 4I, 4S-1D |
| CX17676 | *unc-18; AWC,ASE::unc-18(WT)Rescue; AWA::Chrimson; AIA::GCaMP5A* (line B) | *unc-18(e234)X; kyEx6216* [*ceh-36p::unc-18(WTgenomic)::sl2::tagRFP* 15ng/µl, *unc-122p::GFP* 15ng/µl, *pSM* 70ng/µl]; *kyEx5662; kyEx5128* | 4I, 4S-1D |
| CX17677 | *unc-18; AWC,ASE::unc-18(e234)Sham; AWA::Chrimson; AIA::GCaMP5A* | *unc-18(e234)X; kyEx6218* [*ceh-36p::unc-18(e234genomic)::sl2::tagRFP* 15ng/µl, *unc-122p::GFP* 15ng/µl, *pSM* 70ng/µl]; *kyEx5662; kyEx5128* | 4I, 4S-1D |
| CX17590 | *ASK::GCaMP5A* | *kyEx6191* [*sra-9p::GCaMP5A* 100ng/µl*, elt-2p::mCherry* 2ng/µl] | 5A, 5E, 5S-1A, 5S-1E, 5S-1I, 5S-1L, 5S-3C, 5S-3F, 5S-3J |
| CX17724 | *unc-18; ASK::GCaMP5A* | *unc-18(e234)X; kyEx6191* | 5S-1I, 5S-1L |
| CX17867 | *odr-10; ASK::GCaMP5A* | *odr-10(ky32)X; kyEx6191* | 5S-1I, 5S-1L |
| CX17520 | *AWC::GCaMP5A* | *kyEx6141* [*str-2p::GCaMP5A* 50ng/µl, *unc-122p:dsRed* 15ng/µl] | 5B, 5F, 5S-1B, 5S-1F, 5S-1J, 5S-1M, 5S-3A, 5S-3D, 5S-3GtoJ |
| CX17636 | *unc-18; AWC::GCaMP5A* | *unc-18(e234)X; kyEx6141* | 5S-1J, 5S-1M |
| CX17606 | *odr-10; AWC::GCaMP5A* | *odr-10(ky32)X; kyEx6141* | 5S-1J, 5S-1M |
| CX14571 | *ASE::GCaMP3* | kyEx4732 [*flp-6p::GCaMP3* 5ng/µl, *unc-122p::dsRed* 10ng/µl] | 5C, 5D, 5S-1C, 5S-1G, 5S-1K |
| CX17638 | *unc-18; ASE::GCaMP3* | *unc-18(e234)X; kyEx4732* | 5D, 5S-1K |
| CX16497 | *odr-10; ASE::GCaMP3* | *odr-10(ky32)X; kyEx4732* | 5D, 5S-1K |
| CX10979 | *ASH::GCaMP3* | *kyEx2865* [*sra-6p::GCaMP3* 100ng/µl *ofm-1p::GFP* 10ng/µl] | 5S-1D, 5S-1H |
| CX17751 | *AWA::Chrimson; ASK::GCaMP5A* | *kyEx5662; kyEx6191* | 5S-1N, 5S-1O |
| CX17521 | *AWA::Chrimson; AWC::GCaMP5A* | *kyEx5662; kyEx6141* | 5S-1N, 5S-1O |
| CX17392 | *AWA::Chrimson; ASE::GCaMP3* | *kyEx5662; kyEx4732* | 5S-1N, 5S-1O |
| CX16169 | *ceh-36; AIA::GCaMP5A* | *ceh-36(ky640)X; kyEx5128* | 5ItoK, 5S-3K |
|  | *odr-7 ceh-36; AIA::GCaMP5A* | *odr-7(ky4) ceh-36(ky640)X; kyEx5128* | 5ItoK, 5S-3K |
| CX18038 | *ASK,AWA::GCaMP6s; AIA::GCaMP5A* | *kyIs644* [*che-2p::GCaMP6s* 50ng/µl, *unc-122p::mCherry* 10ng/µl, integrated by UV], *kyEx5128* | 5S-2AtoD |
| CX8293 | *AIA::GFP* | *kyEx1295* [*gcy-28dp::GFP*] | 6A, 6B, 6S-1A, 6S-1B |
| CX16976 | *AIA::GCaMP5A* | *kyIs708* [*kyEx5128*, integrated by UV] | 6AtoC, 6S-1A, 6S-1B |
