# Supplementary material for: Reliability of an interneuron response depends on an integrated sensory state
Source: eLife. 2019 Nov 13;8:e50566. doi: 10.7554/eLife.50566 (PMC6894930; doi:10.7554/eLife.50566)
Supplement: Transparent reporting form [file elife-50566-transrepform.docx]

***eLife’s* transparent reporting form**

We encourage authors to provide detailed information *within their submission* to facilitate the interpretation and replication of experiments. Authors can upload supporting documentation to indicate the use of appropriate reporting guidelines for health-related research (see [EQUATOR Network](http://www.equator-network.org/%20)), life science research (see the [BioSharing Information Resource](https://biosharing.org/)), or the [ARRIVE guidelines](http://www.plosbiology.org/article/info:doi/10.1371/journal.pbio.1000412) for reporting work involving animal research. Where applicable, authors should refer to any relevant reporting standards documents in this form.

If you have any questions, please consult our Journal Policies and/or contact us: [editorial@elifesciences.org](mailto:editorial@elifesciences.org).

**Sample-size estimation**

- You should state whether an appropriate sample size was computed when the study was being designed
- You should state the statistical method of sample size computation and any required assumptions
- If no explicit power analysis was used, you should describe how you decided what sample (replicate) size (number) to use

Please outline where this information can be found within the submission (e.g., sections or figure legends), or explain why this information doesn’t apply to your submission:

We chose our sample sizes based on a preliminary experiment with 24-28 stimulus pulses, described in the Materials and Methods section.

**Replicates**

- You should report how often each experiment was performed
- You should include a definition of biological versus technical replication
- The data obtained should be provided and sufficient information should be provided to indicate the number of independent biological and/or technical replicates
- If you encountered any outliers, you should describe how these were handled
- Criteria for exclusion/inclusion of data should be clearly stated
- High-throughput sequence data should be uploaded before submission, with a private link for reviewers provided (these are available from both GEO and ArrayExpress)

Please outline where this information can be found within the submission (e.g., sections or figure legends), or explain why this information doesn’t apply to your submission:

We discuss and justify delivering two stimulus pulses per animal in the Materials and Methods and in the Results section (shown in Figure 1 – figure supplement 3). We discuss exclusion criteria, rationale for the number of experiments, and the definition of “experiment” versus “experimental block” in Materials and Methods. We show the number of pulses used for analyses in Table S2, Table S3, and Table S4, and in figure legends for Figure 1, Figure 1 – figure supplements 1-3, Figure 3 – figure supplement 1, Figure 5, Figure 5 – figure supplements 1-4, and Figure 6.

**Statistical reporting**

- Statistical analysis methods should be described and justified
- Raw data should be presented in figures whenever informative to do so (typically when N per group is less than 10)
- For each experiment, you should identify the statistical tests used, exact values of N, definitions of center, methods of multiple test correction, and dispersion and precision measures (e.g., mean, median, SD, SEM, confidence intervals; and, for the major substantive results, a measure of effect size (e.g., Pearson's r, Cohen's d)
- Report exact p-values wherever possible alongside the summary statistics and 95% confidence intervals. These should be reported for all key questions and not only when the p-value is less than 0.05.

Please outline where this information can be found within the submission (e.g., sections or figure legends), or explain why this information doesn’t apply to your submission:

We provide the statistical test used, N, all p-values, 95% confidence intervals, mean, standard error of the mean, effect sizes, and the number of statistical comparisons in Table S2, Table S3, and Table S4, with references to figures in the manuscript. For linear regressions, N and R-squared are reported directly in the figures and corresponding legends. We describe each test, as well as which data sets we chose to compare, in the Materials and Methods section. For figures showing magnitudes, we show individual values as well as the median and interquartile range.

(For large datasets, or papers with a very large number of statistical tests, you may upload a single table file with tests, Ns, etc., with reference to sections in the manuscript.)

**Group allocation**

- Indicate how samples were allocated into experimental groups (in the case of clinical studies, please specify allocation to treatment method); if randomization was used, please also state if restricted randomization was applied
- Indicate if masking was used during group allocation, data collection and/or data analysis

Please outline where this information can be found within the submission (e.g., sections or figure legends), or explain why this information doesn’t apply to your submission:

We discuss how groups were allocated, and which experiments used pooled control data, in the Materials and Methods section. We explicitly state that we did not mask groups.

**Additional data files (“source data”)**

- We encourage you to upload relevant additional data files, such as numerical data that are represented as a graph in a figure, or as a summary table
- Where provided, these should be in the most useful format, and they can be uploaded as “Source data” files linked to a main figure or table
- Include model definition files including the full list of parameters used
- Include code used for data analysis (e.g., R, MatLab)
- Avoid stating that data files are “available upon request”

Please indicate the figures or tables for which source data files have been provided:

Source data have been provided for all experiments shown, linked to the corresponding main figure. For all calcium imaging experiments, data are in the form of background-subtracted fluorescence values in labelled summary tables with stimulus timing indicated. For electrophysiology experiments, data are in the form of text files with current or voltage measurements with the protocol indicated. We have provided the analysis code (Matlab) used for all AIA and AWA experiments, which includes functions that can be easily modified for all other analyses using parameters listed in Materials and Methods.
